# Emerging roles of USP7 in tumor immune evasion, metabolic reprogramming, and therapeutic resistance

**DOI:** 10.3389/fimmu.2026.1839581

**Published:** 2026-05-20

**Authors:** Xiaolin Liu, Bo−chi Zhu, Li−li Lian, Yun−feng Li

**Affiliations:** 1 Norman Bethune Second Clinical Medical College, Jilin University, Changchun, Jilin, China; 2 Department of Neurology, The Second Hospital of Jilin University (The Second Norman Bethune Hospital of Jilin University), Changchun, Jilin, China; 3 Department of Radiotherapy, The Second Hospital of Jilin University (The Second Norman Bethune Hospital of Jilin University), Changchun, Jilin, China

**Keywords:** metabolic reprogramming, therapeutic resistance, tumor immune evasion, tumor microenvironment, USP7

## Abstract

Ubiquitin-specific protease 7 (USP7) is a multifunctional deubiquitinase that has emerged as an important regulator of cancer progression, with growing evidence linking it to tumor immune evasion, metabolic adaptation, and therapeutic resistance. USP7 promotes immune suppression by stabilizing checkpoint molecules such as PD-L1, modulating FGL1/LAG-3 signaling, reshaping tumor-associated macrophage polarization, and reinforcing T-cell dysfunction. Simultaneously, USP7 regulates metabolic adaptation by maintaining lipid homeostasis, redox balance, ferroptotic resistance, and nutrient stress responses, thereby supporting tumor survival under adverse conditions. These intertwined immune and metabolic functions collectively contribute to resistance against immune checkpoint blockade, targeted therapy, and other anticancer interventions. Pharmacological inhibition of USP7 has shown promise in reprogramming the tumor microenvironment, exposing metabolic vulnerabilities, and sensitizing tumors to combination therapies. This review summarizes current insights into USP7 structure and substrate networks, highlights its multifaceted roles in tumor immunity and metabolism, and discusses the therapeutic potential and translational challenges of targeting USP7 in cancer.

## Highlights

- USP7 functions as a central hub linking tumor immune suppression, metabolic reprogramming, and therapy resistance.
- USP7 promotes immune escape through checkpoint stabilization, T-cell dysfunction, and macrophage polarization.
- Targeting USP7 exposes metabolic vulnerabilities, reprograms the tumor microenvironment, and enhances immunotherapy efficacy.

## 1 Introduction

Cancer is increasingly understood as a systemic and adaptive disease rather than a disorder driven solely by uncontrolled proliferation. During tumor evolution, malignant cells continuously interact with immune cells, stromal components, extracellular vesicles, and metabolic cues in the tumor microenvironment (TME) (1–3). These interactions enable tumors to survive immune surveillance, adapt to fluctuating nutrient and oxygen availability, and tolerate therapeutic pressure. As a result, tumor immune evasion, metabolic reprogramming, and therapeutic resistance should not be viewed as isolated hallmarks, but as interdependent processes that collectively sustain malignant progression.

Immune escape has become one of the most intensively studied aspects of cancer biology, particularly after the clinical success of immune checkpoint blockade (4–6). However, despite impressive responses in selected patient populations, a substantial proportion of tumors remain intrinsically resistant or acquire resistance during treatment. This limitation has highlighted a key concept in cancer immunology: immune suppression is rarely mediated by a single molecule or pathway. Instead, it emerges from coordinated regulation of checkpoint molecules, cytokine and chemokine networks, myeloid-cell polarization, T-cell functional exhaustion, angiogenesis, and stromal remodeling. In parallel, tumor metabolism has been recognized as a critical determinant of immune responsiveness. Cancer cells compete with immune cells for nutrients, remodel lipid and redox homeostasis, and generate metabolites that reinforce immunosuppressive niches. Thus, the crosstalk between metabolism and immunity has become central to understanding why some tumors are refractory to therapy.

At the molecular level, many of these adaptive programs are controlled by post-translational modifications. Among them, ubiquitination is one of the most versatile mechanisms for regulating protein stability, localization, activity, and signaling output. The ubiquitin–proteasome system is balanced by ubiquitin ligases and deubiquitinases (DUBs), and dysregulation of either arm can profoundly influence tumor biology (7–11). DUBs are particularly attractive because they often function as signaling rheostats rather than simple binary switches. By reversing ubiquitin conjugation, they fine-tune the abundance and persistence of oncogenic and immunoregulatory proteins, thereby shaping dynamic cellular responses to stress.

USP7, also known as HAUSP, is one of the best-characterized members of the DUB family. Historically, USP7 was first appreciated for its role in the p53–MDM2 axis, where it regulates a classical tumor suppressor pathway (12–16). Over time, however, the biological scope of USP7 has expanded dramatically. It is now clear that USP7 controls multiple substrates involved in epigenetic remodeling, transcriptional regulation, DNA damage responses, oxidative stress adaptation, inflammatory signaling, and immune escape. Recent studies have further revealed that USP7 is not only a tumor-cell-intrinsic regulator but also a critical determinant of the tumor microenvironment. It can stabilize programmed death-ligand 1 (PD-L1), enhance fibrinogen-like protein 1 (FGL1)/lymphocyte activation gene 3 (LAG-3)-mediated immunosuppression, reshape tumor-associated macrophage (TAM) polarization, influence fibroblast-derived angiogenic programs, and promote T-cell dysfunction. At the same time, USP7 is increasingly linked to lipid metabolism, ferroptosis susceptibility, antioxidant pathways, and nutrient-stress signaling, placing it at the intersection of metabolism and immunity. These emerging findings have important translational implications. First, they suggest that USP7 may serve as a mechanistic bridge between tumor intrinsic oncogenic signaling and extrinsic immune suppression (17). Second, they indicate that therapeutic targeting of USP7 could generate broader effects than inhibition of a single immune checkpoint or metabolic enzyme. Third, they raise the possibility that USP7 may be particularly relevant in tumors characterized by immune exclusion, metabolic plasticity, and poor response to conventional immunotherapy (18). Accordingly, a comprehensive understanding of USP7 is needed not only from a mechanistic standpoint but also from the perspective of treatment design and patient stratification.

In this review, we do not aim to provide another broad summary of canonical USP7 biology centered primarily on the p53/MDM2 axis. Instead, we specifically position USP7 as a context-responsive mechanistic node that links tumor-intrinsic survival programs with extrinsic microenvironmental regulation. We highlight how USP7 integrates immune checkpoint stabilization, macrophage and T-cell dysfunction, metabolic adaptation, and therapy resistance into an interconnected framework of malignant fitness. By organizing the literature through this immunity–metabolism–resistance axis, this review offers a more conceptually unified perspective on USP7 and provides a rationale for biomarker-guided and combination-based therapeutic strategies.

## 2 Biological basis of USP7 in cancer

### 2.1 Structure and catalytic mechanism of USP7

USP7 is a cysteine protease belonging to the ubiquitin-specific protease family and possesses a multidomain architecture that underlies its broad substrate selectivity. The N-terminal TRAF-like domain is important for substrate docking and often recognizes short consensus peptide motifs. The central catalytic domain contains the canonical catalytic triad required for deubiquitinating activity, while the C-terminal ubiquitin-like (UBL) domains modulate substrate binding, catalytic activation, and allosteric regulation (19–23). This structural organization allows USP7 to engage a wide variety of proteins in a highly context-dependent manner.

An important feature of USP7 biology is that its catalytic efficiency is not fixed. Instead, substrate recognition and enzymatic activity are shaped by conformational transitions, binding partners, subcellular localization, and post-translational modifications (24). This may explain USP7 can exert different biological effects in distinct tumor settings. In one context, USP7 may predominantly stabilize proteins that support oncogenic signaling and immune suppression; in another, it may act through alternative substrate networks with different consequences. Thus, the enzymatic plasticity of USP7 is central to its biological complexity. From a therapeutic perspective, the structural domains of USP7 also provide multiple opportunities for pharmacological intervention. Small molecules may inhibit the catalytic domain directly, disrupt allosteric activation, or interfere with protein–protein interactions required for substrate recognition (25). This has stimulated intensive drug-discovery efforts aimed at developing selective USP7 inhibitors with improved potency and translational potential.

### 2.2 Major substrates and signaling networks regulated by USP7

The best-known substrate network of USP7 involves the p53–MDM2 axis. By deubiquitinating MDM2 and, in some contexts, p53 itself, USP7 influences the balance between tumor suppressor activity and oncogenic stress tolerance (26–30). However, it has become increasingly clear that the biological significance of USP7 extends far beyond this classical pathway. USP7 regulates multiple proteins involved in transcriptional control, chromatin remodeling, DNA damage responses, oxidative stress adaptation, immune suppression, and therapy resistance.

Among the many reported substrates, several are directly relevant to the themes of this review. USP7 can stabilize programmed death-ligand 1 (PD-L1), a key immune checkpoint ligand that suppresses antitumor T-cell activity, thereby promoting tumor immune escape. It can also influence enhancer of zeste homolog 2 (EZH2)-dependent epigenetic programs. EZH2 is the catalytic subunit of Polycomb repressive complex 2 (PRC2) and mediates transcriptional repression through histone methylation, which in turn can affect immune checkpoint expression and malignant progression (31–33). In some contexts, USP7 regulates TRIM24, a multifunctional co-regulator linked to transcriptional control and macrophage polarization. USP7 has also been implicated in maintaining NRF2 signaling, connecting deubiquitination to redox homeostasis and oxidative stress adaptation. More recently, the USP7–SCD axis has linked this DUB to lipid metabolism and ferroptosis resistance. In liver cancer, USP7 contributes to the PRDM1/FGL1 pathway, thereby expanding its relevance to non-PD-L1 checkpoint-associated immune suppression. The diversity of USP7 substrates indicates that this enzyme is not confined to a single pathway but acts as a molecular organizer across several adaptive signaling systems. This property may explain why USP7 inhibition often has pleiotropic effects, simultaneously altering tumor proliferation, metabolism, immune evasion, and therapeutic sensitivity. It also implies that the functional consequences of USP7 targeting will vary according to the dominant substrate landscape of a given cancer type.

### 2.3 Aberrant expression and clinical significance across cancers

USP7 is aberrantly expressed in a broad spectrum of tumors, including lung cancer, gastric cancer, hepatocellular carcinoma, colorectal cancer, glioma, cervical cancer, breast cancer, and nasopharyngeal carcinoma (34). In many studies, elevated USP7 expression correlates with more aggressive clinicopathological behavior, increased immune suppression, and poorer clinical outcome. These associations support the view that USP7 frequently acts as a tumor-promoting factor. Yet the prognostic and biological meaning of USP7 should not be oversimplified. First, USP7 expression does not automatically indicate dependence on the same substrates in all tumors. For instance, in one cancer, the dominant output of USP7 may be checkpoint stabilization, whereas in another it may be metabolic adaptation or stromal remodeling. Second, the cellular compartment in which USP7 acts is critical. Tumor-cell-intrinsic USP7 may enhance immune escape, but USP7 activity in macrophages or other stromal populations may generate additional, sometimes contrasting, effects (35, 36). Third, tumor lineage, genetic background, oxygen tension, nutrient availability, and therapy exposure may all shift the substrate preference and biological output of USP7.

**Figure 1:**
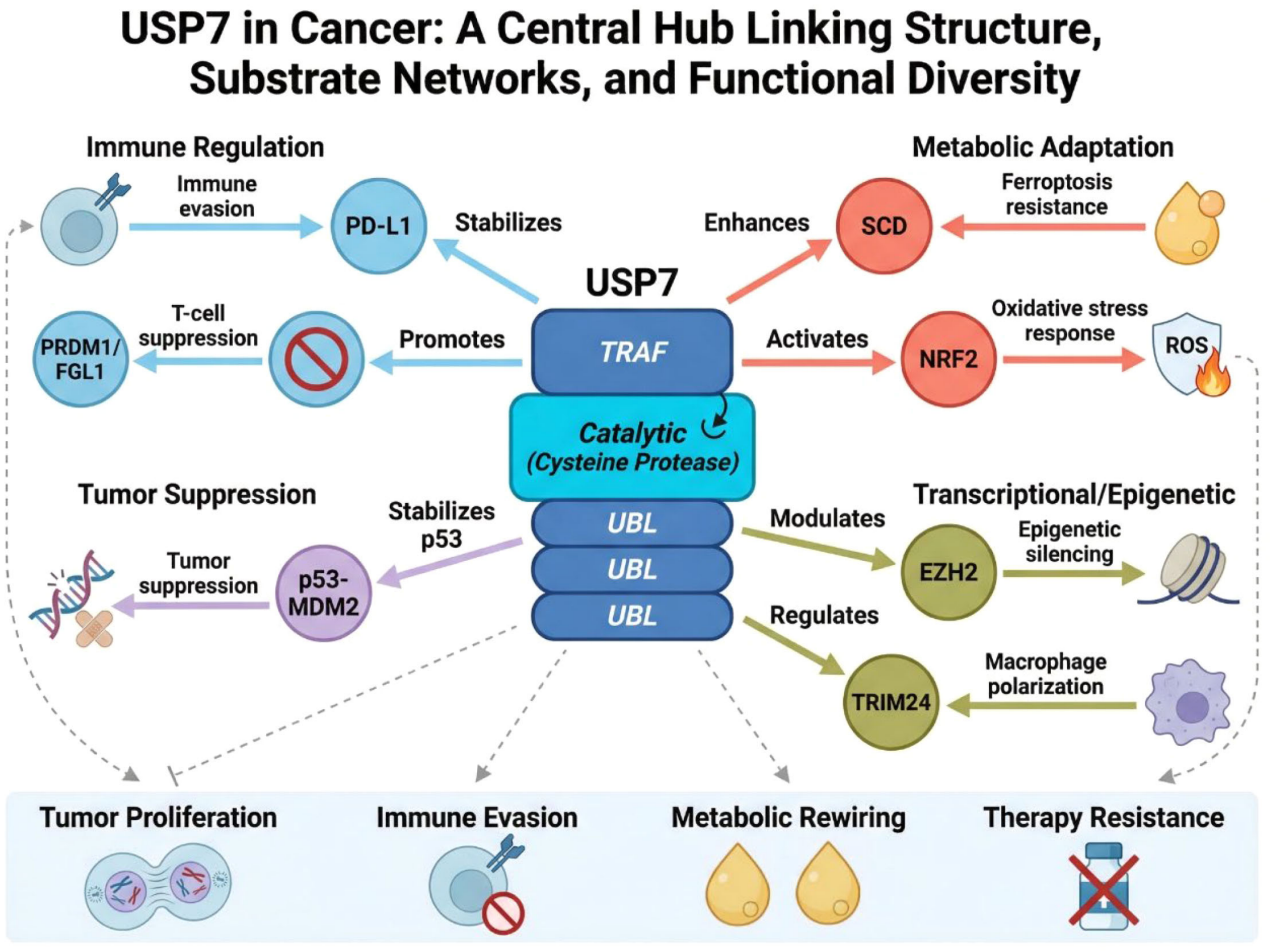
Biological basis of USP7 in cancer. This schematic illustrates the multidomain architecture of USP7, including the N-terminal TRAF-like domain, central catalytic domain, and C-terminal UBL domains, and summarizes its major substrate networks. By regulating proteins such as p53/MDM2, PD-L1, EZH2, TRIM24, NRF2, SCD, and PRDM1/FGL1, USP7 coordinates oncogenic signaling, immune evasion, metabolic adaptation, and therapeutic resistance in a context-dependent manner.

These context-dependent features are especially important when considering USP7 as a therapeutic target. Rather than assuming a uniform oncogenic role, it may be more appropriate to regard USP7 as a context-responsive regulator of malignant fitness. This framework better accommodates the apparently divergent observations reported in different tumor types and may help explain why USP7 inhibition can have both direct tumoricidal and microenvironmental effects. **Figure 1** summarizes the structural basis and major substrate networks of USP7 in cancer. It highlights USP7 as a context-dependent molecular hub that integrates immune suppression, metabolic adaptation, and malignant progression through coordinated deubiquitination of multiple oncogenic and immunoregulatory targets.

## 3 USP7 and tumor immune evasion

### 3.1 USP7 stabilizes immune checkpoint signaling to promote immune escape

One of the most direct mechanisms by which USP7 promotes immune evasion is through the stabilization of immune checkpoint-related proteins. PD-L1 is a particularly important example. In multiple tumor contexts, PD-L1 functions as a key suppressor of antitumor immunity by binding PD-1 on T cells and dampening cytotoxic responses. While PD-L1 has often been studied at the transcriptional level, it is now evident that its protein stability is also tightly regulated by ubiquitination. In gastric cancer, USP7 directly interacts with PD-L1 and removes ubiquitin chains that would otherwise target the protein for degradation (37). As a consequence, PD-L1 accumulates on tumor cells, strengthening immune escape and reducing the susceptibility of cancer cells to T-cell-mediated killing.

A similar regulatory logic has been observed in glioma, further supporting the notion that USP7-mediated PD-L1 stabilization is not restricted to a single tumor type (38). These findings are highly significant because they place USP7 upstream of a clinically actionable checkpoint pathway. Rather than merely reflecting increased PD-L1 transcription, elevated checkpoint signaling in these tumors may depend on a post-translational mechanism that prolongs PD-L1 persistence and thereby sustains immunosuppression. This has therapeutic implications, as it suggests that USP7 inhibition could reduce PD-L1 protein abundance even in settings where upstream inflammatory signaling remains active.

**Figure 2:**
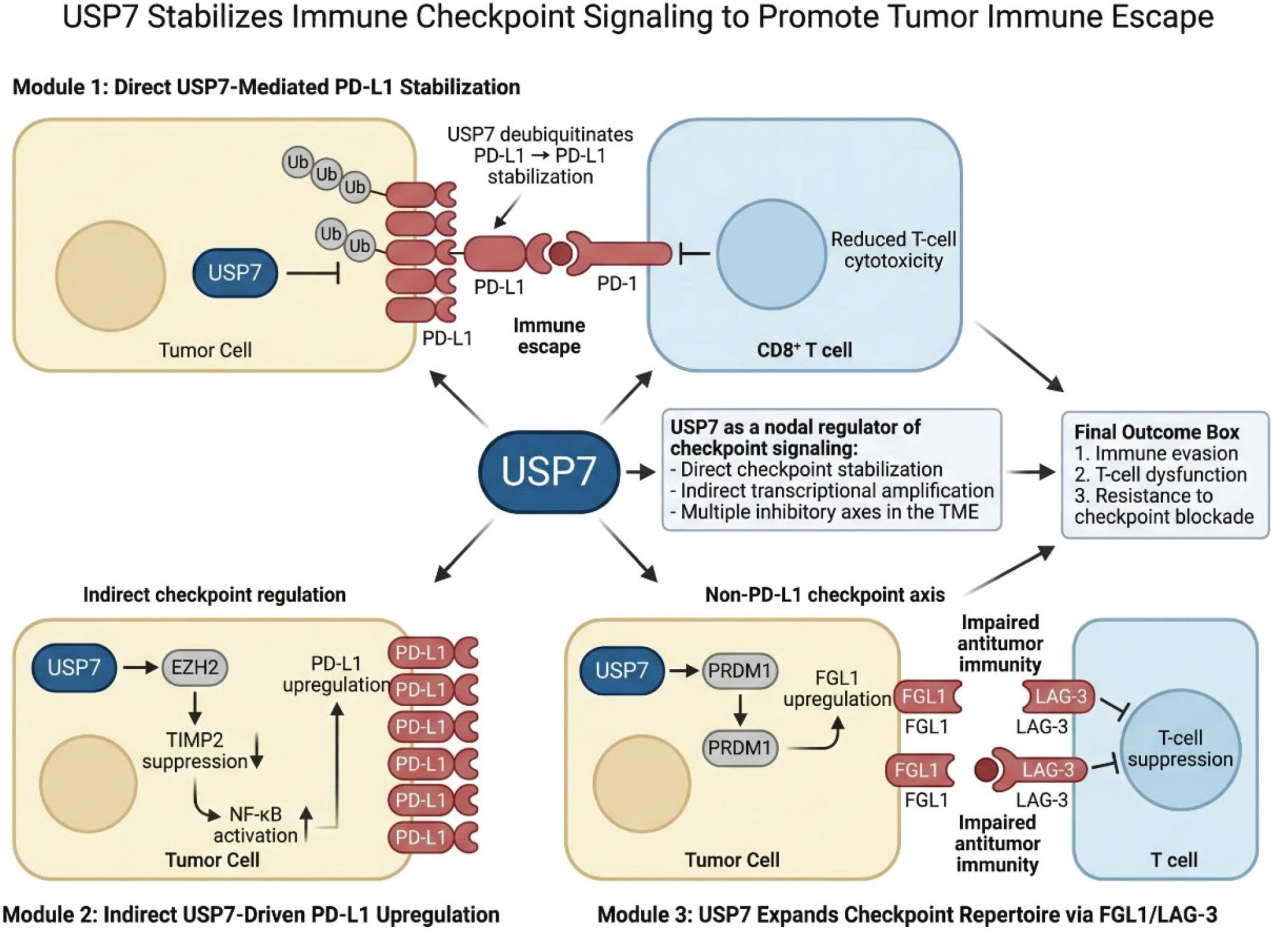
USP7 stabilizes immune checkpoint signaling to promote immune escape. This schematic shows how USP7 promotes tumor immune evasion through direct deubiquitination and stabilization of PD-L1, indirect activation of the EZH2/TIMP2/NF-κB/PD-L1 axis, and enhancement of the PRDM1/FGL1/LAG-3 pathway. Together, these mechanisms position USP7 as a nodal regulator of checkpoint signaling and T-cell suppression in the tumor microenvironment.

Importantly, USP7 can also regulate checkpoint biology through indirect mechanisms. In cervical cancer, USP7 enhances the EZH2/TIMP2/NF-κB/PD-L1 axis, indicating that it can drive checkpoint expression via epigenetic and inflammatory signaling pathways (39). This distinction between direct deubiquitination and indirect pathway modulation is conceptually important. It suggests that USP7 acts not only as a stabilizer of immune checkpoint proteins but also as a broader amplifier of immunosuppressive transcriptional programs. Such dual regulation may contribute to the robustness of immune evasion and partly explain why targeting a single downstream checkpoint is not always sufficient. The immunological scope of USP7 extends beyond PD-L1. In hepatocellular carcinoma, USP7 promotes PRDM1-mediated FGL1 upregulation, thereby engaging the FGL1/LAG-3 axis, which is increasingly recognized as an important mediator of T-cell suppression (40). This finding broadens the checkpoint repertoire of USP7 and indicates that it may contribute to resistance not only to PD-1/PD-L1 blockade but also to emerging checkpoint-directed therapies. Overall, these data position USP7 as a nodal regulator of checkpoint biology, capable of coordinating multiple inhibitory axes within the tumor microenvironment. **Figure 2** summarizes how USP7 amplifies immune checkpoint signaling through both direct and indirect pathways. It highlights USP7 as a central regulator of tumor immune escape by coordinating PD-L1- and FGL1/LAG-3-dependent suppression of antitumor T-cell responses.

### 3.2 USP7 reshapes the tumor immune microenvironment through macrophage polarization

Macrophages are among the most functionally versatile cells in the tumor microenvironment. Depending on the signals they receive, they can acquire antitumor M1-like features or immunosuppressive, tissue-remodeling M2-like characteristics (41–45). Because TAMs influence antigen presentation, cytokine production, angiogenesis, extracellular matrix remodeling, and T-cell activity, mechanisms that control their polarization are of major importance in cancer progression and treatment response. USP7 has emerged as one such mechanism. In lung cancer, pharmacological inhibition of USP7 was shown to reprogram TAMs from an M2-like phenotype toward an M1-like state (46). This shift was associated with activation of p38 MAPK signaling, enhanced cytotoxic T-lymphocyte activity, and improved antitumor immune responses. Notably, macrophage depletion attenuated the antitumor effect of USP7 inhibition, indicating that its therapeutic benefit was not solely tumor cell-intrinsic but depended substantially on microenvironmental reprogramming. These findings support the concept that USP7 inhibition can convert a suppressive myeloid milieu into one that is more permissive to effective antitumor immunity.

However, the relationship between USP7 and macrophage polarization is not strictly linear. In breast cancer, tumor-derived exosomal circ-0100519 promotes M2 macrophage polarization through the USP7/NRF2 axis (47). Here, USP7 acts downstream of intercellular communication mediated by extracellular vesicles, linking tumor-secreted noncoding RNA signals to antioxidant adaptation and myeloid reprogramming. This axis illustrates how USP7 can function as a conduit through which tumor cells educate surrounding macrophages to support tumor growth and immune suppression. In contrast, work in nasopharyngeal carcinoma suggests that USP7 may inhibit tumor progression by promoting SPLUNC1-mediated M1 polarization through TRIM24 (48). Although this finding appears paradoxical relative to the more common pro-tumor role of USP7, it is highly informative. It indicates that the biological outcome of USP7 activity depends on substrate context, lineage-specific transcriptional networks, and the composition of the local microenvironment. Rather than invalidating the idea that USP7 promotes immune suppression, such context dependence underscores the need for more refined interpretation. It may be that in some tumors USP7 primarily stabilizes immunosuppressive factors, whereas in others it preserves proteins that favor inflammatory differentiation.

**Figure 3:**
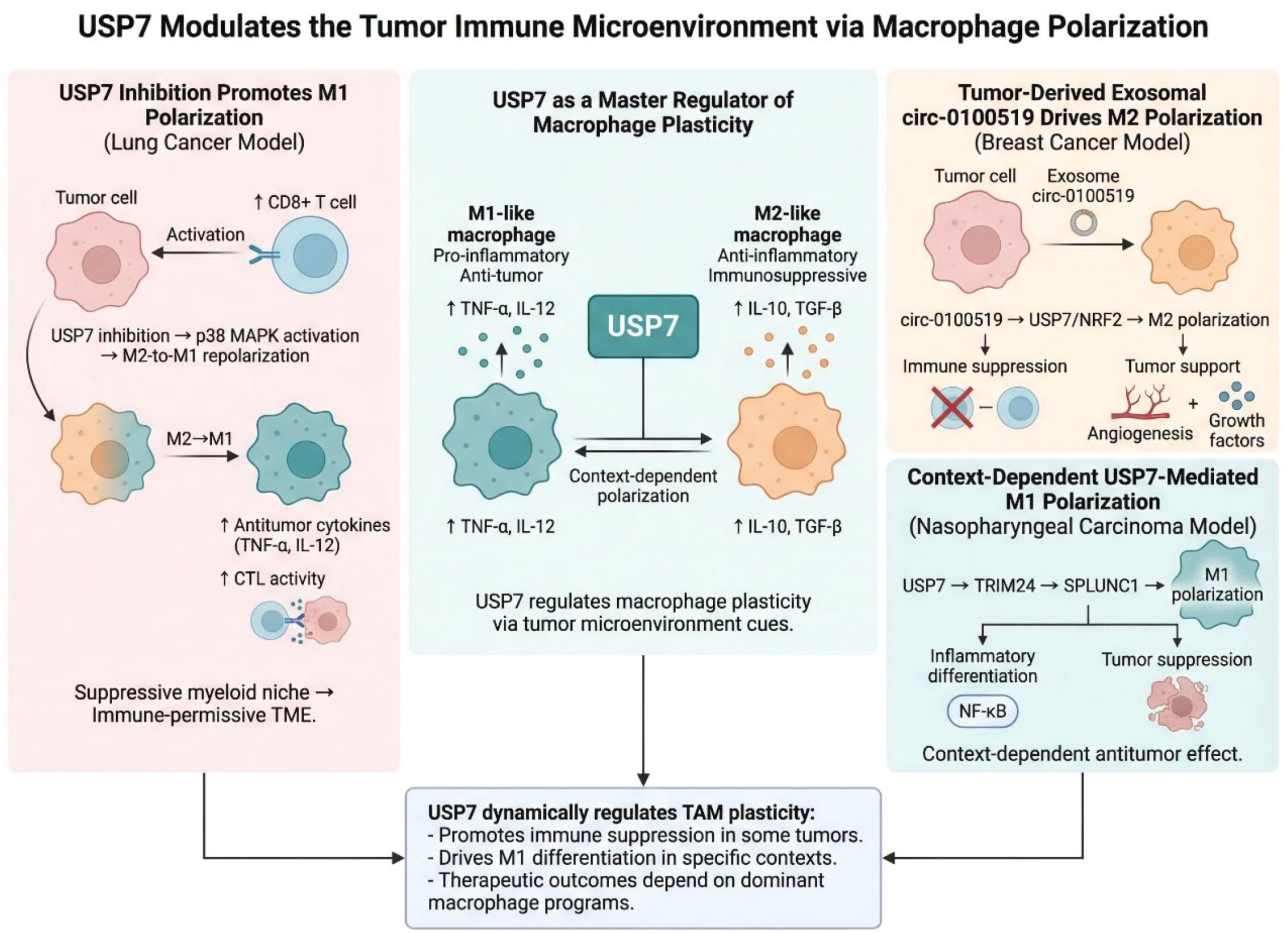
USP7 reshapes the tumor immune microenvironment through macrophage polarization. This schematic illustrates how USP7 regulates tumor-associated macrophage plasticity in a context-dependent manner. USP7 inhibition promotes M2-to-M1 repolarization and antitumor immunity, whereas tumor-derived exosomal circ-0100519 drives M2 polarization through the USP7/NRF2 axis. In specific settings, USP7 may also support M1 polarization through TRIM24/SPLUNC1 signaling.

Taken together, these studies suggest that USP7 functions as a regulator of macrophage plasticity rather than as a uniform promoter of any single polarization state. This context dependence has important implications for therapeutic design. For clinical translation of USP7-targeted strategies, it will be essential to define not only USP7 expression, but also the dominant macrophage-associated programs operating within each tumor ecosystem (**Figure 3**).

### 3.3 USP7 regulates T-cell activity, dysfunction, and exhaustion

While checkpoint ligands and macrophages are major components of immune suppression, the functional state of T cells ultimately determines whether an effective antitumor response can be mounted (49). Cytotoxic CD8^+ T cells are frequently impaired in tumors through chronic antigen stimulation, nutrient deprivation, checkpoint signaling, and transcriptional reprogramming toward exhaustion. Recent studies indicate that USP7 contributes to several of these processes. In hepatocellular carcinoma, USP7 inhibition enhances CD8^+ T-cell activity by suppressing PRDM1-mediated FGL1 upregulation. This finding is important because it demonstrates that the immunological impact of USP7 is not limited to checkpoint stabilization on tumor cells, but extends to the functional quality of effector T-cell responses. By reducing FGL1-dependent inhibitory signaling, USP7 blockade allows CD8^+ T cells to recover antitumor capacity. An even more conceptually rich example comes from studies of short-term starvation in liver cancer, where metabolic stress was found to inhibit CD36 N-glycosylation, activate AMPK, and reduce USP7 UFMylation. The resulting destabilization of RBPJ attenuated IRF4/TNFRSF1B-associated exhaustion programs in T cells. This work places USP7 within a broader network linking nutrient sensing, post-translational modification, transcriptional control, and T-cell exhaustion. The implication is that USP7 is not only a determinant of immune suppression but also a targetable node through which metabolic interventions may reprogram antitumor immunity.

**Figure 4:**
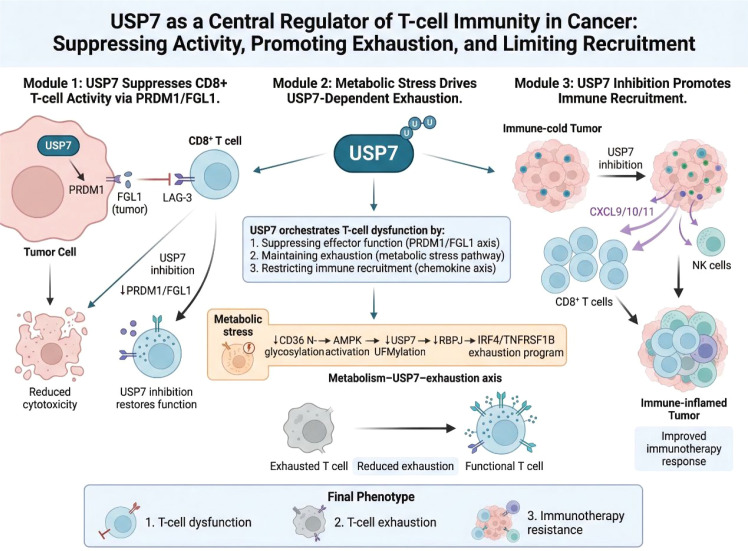
USP7 regulates T-cell activity, dysfunction, and exhaustion in cancer. This schematic shows how USP7 suppresses antitumor T-cell immunity by enhancing PRDM1/FGL1-mediated inhibitory signaling, sustaining metabolic stress-associated exhaustion programs, and limiting chemokine-driven recruitment of CD8^+ T cells and NK cells. Together, these mechanisms position USP7 as a central regulator of T-cell dysfunction and immunotherapy resistance.

Beyond specific ligand-receptor systems, USP7 also appears to influence the broader immune composition of tumors. In microsatellite-stable colorectal cancer, USP7 inhibition enhances chemokine programs that promote recruitment of CD8^+ T cells and NK cells (50, 51). Thus, USP7 may affect T-cell biology both qualitatively and quantitatively: it can suppress the function of infiltrating T cells and also contribute to the failure of their recruitment into tumors. This dual influence is highly relevant to resistance against checkpoint blockade, as successful immunotherapy requires both sufficient immune infiltration and preservation of effector function (**Figure 4**).

### 3.4 USP7 promotes immune-excluded and immune-cold tumor ecosystems

The concepts of “hot” and “cold” tumors have become central to immuno-oncology. Tumors with abundant cytotoxic lymphocyte infiltration and active inflammatory signaling are generally more responsive to immunotherapy, whereas tumors lacking immune infiltration are often resistant (52–54). The molecular determinants of this distinction remain incompletely understood, but recent data suggest that USP7 is one such determinant.

In microsatellite-stable colorectal cancer, an immunotherapy-refractory subtype, USP7 was found to be associated with an immune-cold microenvironment (55). Targeting USP7 enhanced expression of chemokines such as CXCL9, CXCL10, and CXCL11, increased infiltration by CD8^+ T cells and NK cells, and improved responsiveness to anti-PD-1 therapy. These effects indicate that USP7 contributes not merely to local T-cell inhibition but to the larger architectural organization of immune exclusion. By suppressing inflammatory recruitment signals and maintaining an unfavorable microenvironment, USP7 helps preserve a state of primary resistance. This concept can be extended to other tumors in which USP7 influences macrophage polarization, checkpoint ligand abundance, stromal VEGF production, or T-cell exhaustion. Together, these mechanisms suggest that USP7 functions as a systems-level coordinator of immune hostility. It does not simply block one arm of the immune response; rather, it reinforces several interconnected features of immune exclusion, including myeloid suppression, vascular barriers, inadequate chemokine support, and persistent inhibitory signaling. This broader framing may better capture why USP7 inhibition can synergize with immune checkpoint blockade even in tumors with poor baseline responsiveness (56). **Table 1** highlights USP7’s immune regulatory functions in various cancers, emphasizing substrate/pathway, immune consequences, and translational relevance.

**Table 1:** Overview of USP7-driven immune modulation across cancer types.

Cancer type	Experimental model	USP7-regulated target/pathway	Immune effect	Main findings/implications
Lung cancer	Cell lines, murine models	PD-L1, TAMs	Promotes M2→M1 reprogramming, enhances CTL activity	USP7 inhibition remodels TAMs, synergizes with anti-PD-1 therapy
Gastric cancer	Cell lines, patient samples	PD-L1	Immune checkpoint stabilization	USP7 inhibition reduces PD-L1, sensitizes tumor cells to T-cell killing
Hepatocellular carcinoma	Murine models	FGL1/LAG-3	CD8^+^ T cell activity suppression	USP7 blockade restores T-cell cytotoxicity
Colorectal cancer (MSS)	Cell lines, murine models	CXCL9/10/11	Cold-to-hot tumor conversion	USP7 inhibition recruits CD8^+^ T/NK cells, enhances anti-PD-1 efficacy
Breast cancer	Exosome co-culture	NRF2, M2 macrophage polarization	Immunosuppression via M2 TAM	Tumor-derived circRNA uses USP7/NRF2 axis to polarize macrophages
Nasopharyngeal carcinoma	Cell lines, murine models	TRIM24, SPLUNC1	Promotes M1 polarization	USP7 supports antitumor macrophage phenotype (context-dependent)
Glioma	Cell lines	PD-L1	Immune escape	USP7 stabilizes PD-L1, enhances tumor evasion

## 4 USP7 and metabolic reprogramming in cancer

### 4.1 USP7 as a regulator of metabolic plasticity

Metabolic reprogramming is an essential feature of malignant progression. Cancer cells must adapt to fluctuating oxygen levels, nutrient limitation, oxidative stress, and therapeutic insult while maintaining biosynthesis, redox balance, and survival signaling. Although USP7 is not itself a classical metabolic enzyme, recent evidence indicates that it regulates metabolic plasticity by controlling the stability of proteins that govern lipid metabolism, antioxidant defense, and stress adaptation (57–59). In this sense, USP7 serves as an upstream molecular coordinator of metabolic state rather than a direct catalyst of metabolic reactions. This distinction is important. By targeting central regulators rather than metabolites themselves, USP7 can influence multiple metabolic outputs simultaneously. Such regulation may be particularly advantageous for tumors, as it permits dynamic adaptation to environmental stress without requiring permanent genetic rewiring. From a translational standpoint, this also means that USP7 inhibition may expose latent metabolic vulnerabilities that are otherwise buffered by adaptive signaling.

### 4.2 USP7 controls lipid metabolism and ferroptotic susceptibility

**Figure 5:**
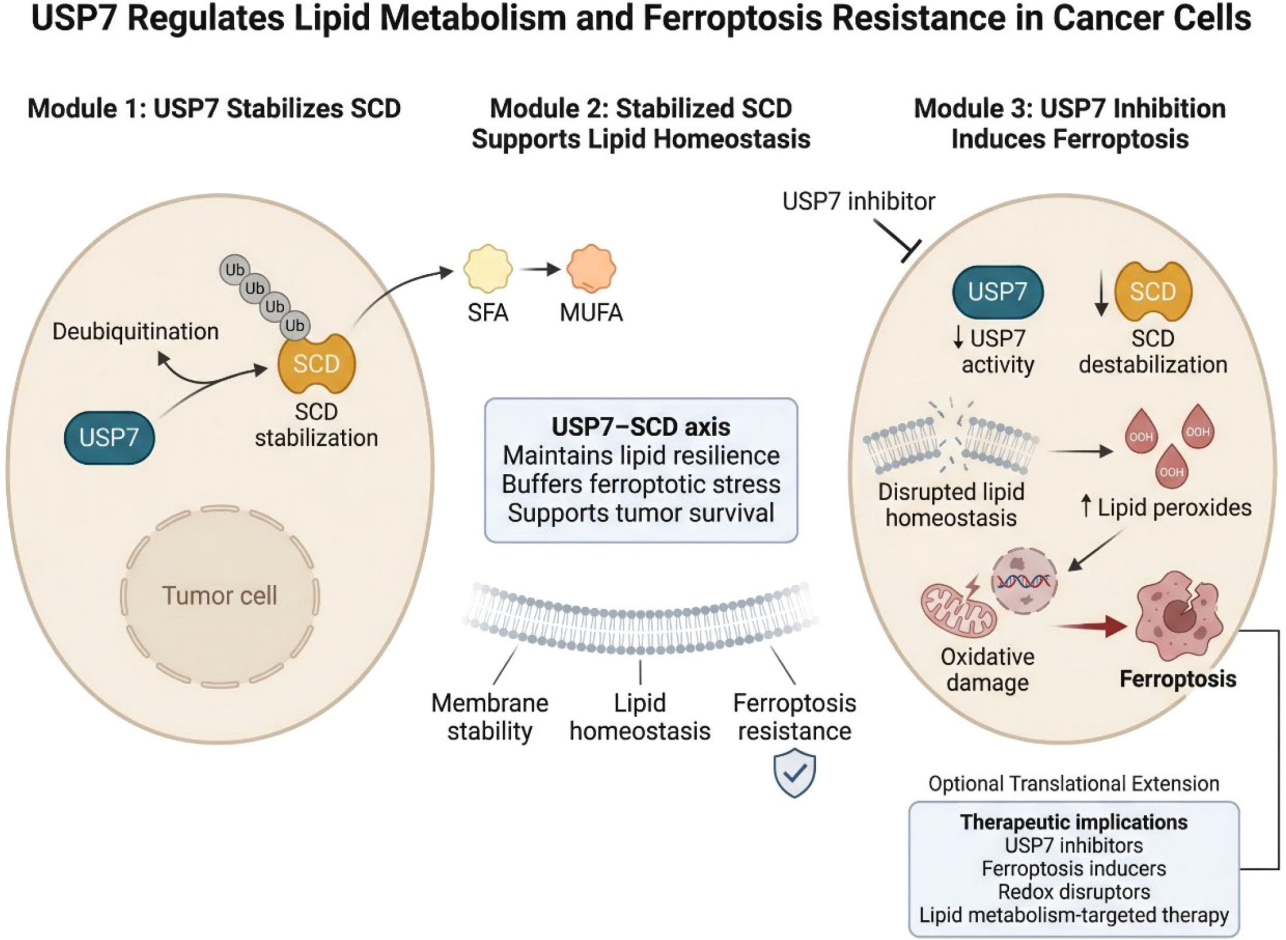
USP7 controls lipid metabolism and ferroptotic susceptibility through the USP7–SCD axis. This schematic illustrates how USP7 stabilizes SCD to maintain lipid homeostasis, membrane integrity, and resistance to lipid peroxidation. In contrast, USP7 inhibition destabilizes SCD, disrupts lipid metabolic resilience, promotes lipid peroxide accumulation, and induces ferroptosis, thereby exposing a metabolic vulnerability in cancer cells.

Among the most direct links between USP7 and metabolism is the recently identified USP7–SCD axis in gastric cancer. Stearoyl-CoA desaturase (SCD) is a key enzyme in lipid metabolism that catalyzes the conversion of saturated fatty acids into monounsaturated fatty acids (60–65). This function is critical for membrane fluidity, lipid signaling, and resistance to oxidative lipid damage. By stabilizing SCD, USP7 supports a lipid-metabolic state favorable to tumor survival. When USP7 is inhibited, SCD becomes destabilized, leading to disrupted lipid homeostasis, accumulation of lipid peroxides, and induction of ferroptosis. This is a major conceptual advance because it links deubiquitination to an emerging form of regulated cell death that is tightly coupled to metabolic state. It also suggests that one function of USP7 in cancer is to buffer against ferroptotic stress by preserving enzymes that maintain lipid resilience. The implications go beyond gastric cancer (66). Lipid remodeling is a widespread adaptive mechanism in cancer, especially in therapy-resistant tumors exposed to oxidative damage (67–69). If USP7 broadly contributes to maintaining lipid homeostasis, then its inhibition may provide a strategy for collapsing metabolic defenses in tumors that otherwise tolerate oxidative and therapeutic stress. This raises the possibility of combining USP7 inhibition with ferroptosis inducers, redox disruptors, or lipid metabolism-targeted therapies (**Figure 5**).

### 4.3 USP7, redox homeostasis, and oxidative stress adaptation

Another crucial aspect of tumor metabolism is the management of oxidative stress (70–74). Cancer cells often exist in a state of elevated reactive oxygen species (ROS) production due to rapid proliferation, mitochondrial dysfunction, oncogenic signaling, and therapy exposure. To survive, they must activate antioxidant and detoxification programs. NRF2 is a master regulator of this response, and recent work indicates that USP7 can reinforce NRF2-related signaling in an immunologically relevant context.

**Figure 6:**
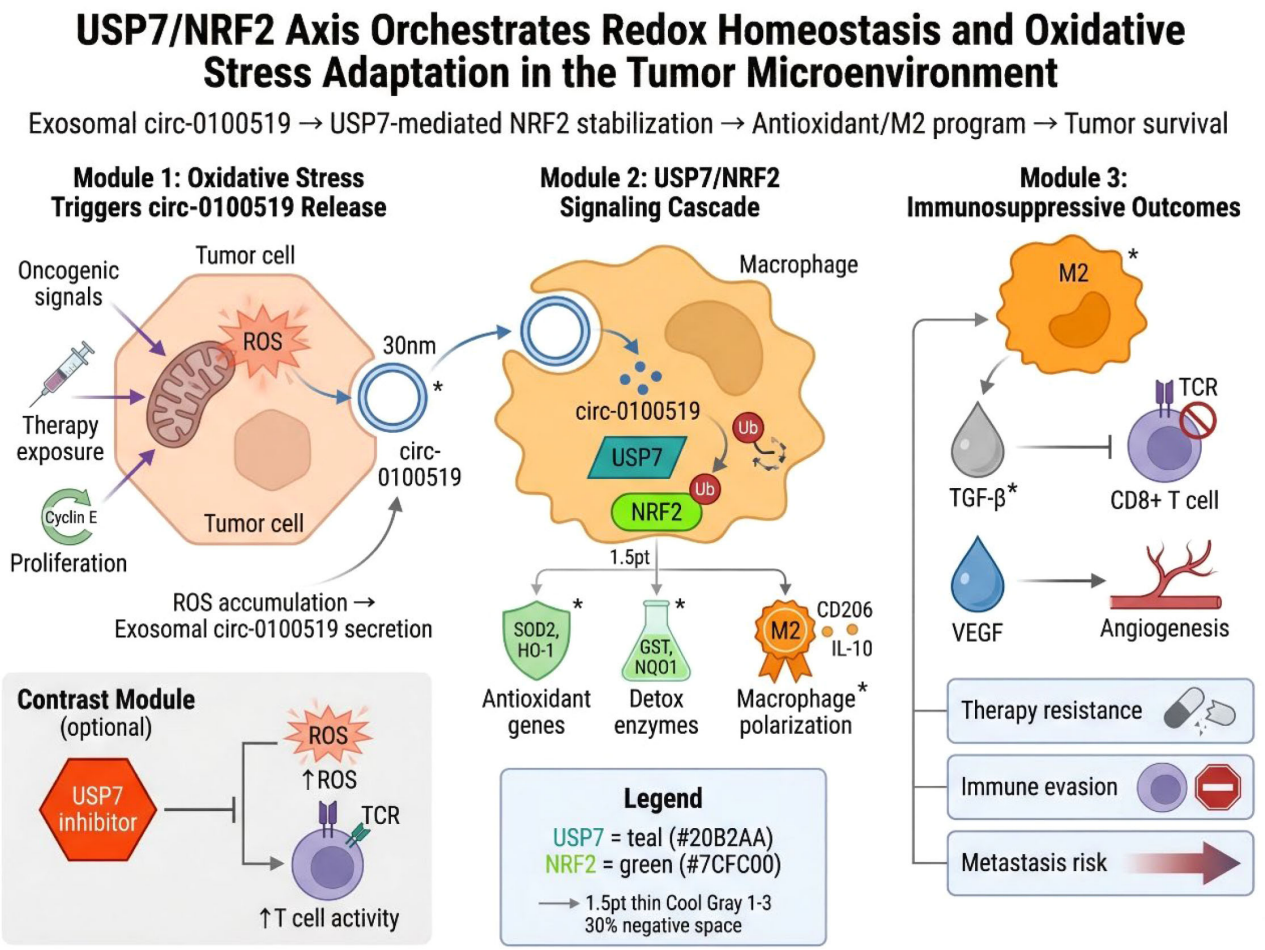
USP7 reinforces redox homeostasis and oxidative stress adaptation through the USP7/NRF2 axis. This schematic illustrates how tumor-derived exosomal circ-0100519 activates the USP7/NRF2 axis, enhances antioxidant programs, and promotes M2 macrophage polarization. Through this pathway, USP7 couples oxidative stress adaptation to immune suppression, angiogenesis, and treatment resistance, thereby supporting tumor progression.

In breast cancer, exosomal circ-0100519 promotes M2 macrophage polarization through the USP7/NRF2 axis. This suggests that USP7-mediated stabilization of antioxidant programs does not merely protect tumor cells from oxidative injury; it may also alter the immune microenvironment in favor of tumor progression. M2 macrophages are often associated with tissue repair, angiogenesis, and immune suppression, and redox balance can influence their differentiation and function (75). Thus, the USP7/NRF2 connection highlights an important principle: metabolic adaptation and immune suppression are often co-regulated rather than separable phenomena. This insight may have broader significance. Tumors that maintain strong antioxidant capacity often resist both chemotherapy and immune-mediated stress. If USP7 helps sustain these programs, then its inhibition may weaken redox defenses in ways that sensitize tumors to treatment while also reshaping immune-cell behavior. Such dual effects are especially attractive in tumors where metabolic adaptation and immune escape are tightly intertwined. **Figure 6** summarizes how the USP7/NRF2 axis links redox homeostasis to immune remodeling in the tumor microenvironment. It highlights that USP7-driven antioxidant adaptation not only protects against oxidative stress but also promotes M2 macrophage polarization and immunosuppressive tumor progression.

### 4.4 Nutrient stress, post-translational modification, and metabolism–immune crosstalk

**Figure 7:**
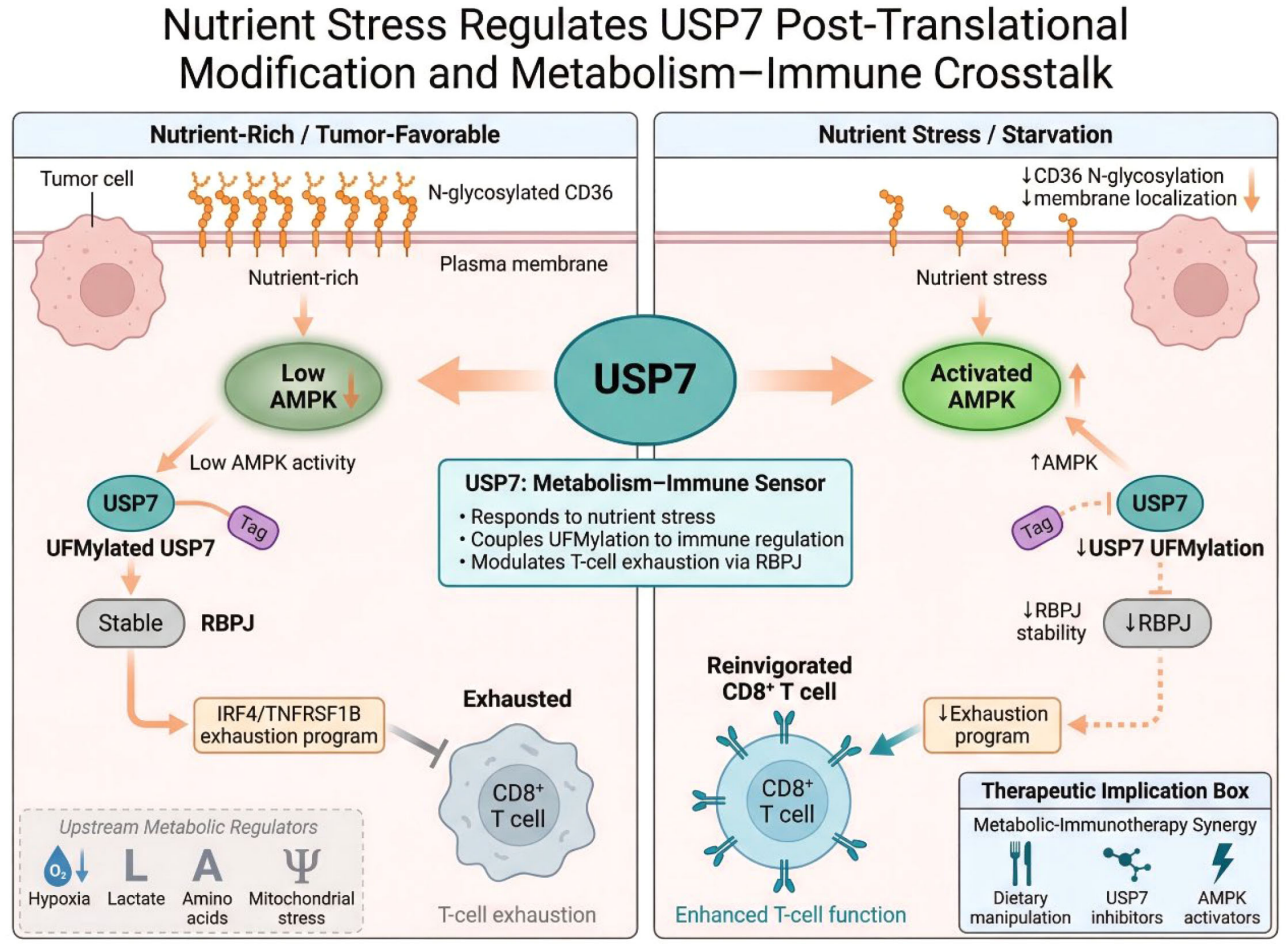
Nutrient stress regulates USP7 post-translational modification and metabolism–immune crosstalk. This schematic illustrates how nutrient stress reduces CD36 N-glycosylation, activates AMPK, and suppresses USP7 UFMylation, leading to RBPJ destabilization and attenuation of the IRF4/TNFRSF1B-associated T-cell exhaustion program. These findings position USP7 as a metabolically responsive node linking environmental stress to immune reprogramming.

Metabolic regulation of USP7 is not one-directional. In addition to controlling metabolic adaptation, USP7 can itself be modified by metabolic context. Short-term starvation studies in liver cancer showed that nutrient stress affects CD36 N-glycosylation, membrane localization, and AMPK activation, which in turn reduces USP7 UFMylation (76). This regulatory cascade ultimately destabilizes RBPJ and alleviates T-cell exhaustion. These results are notable because they place USP7 downstream of metabolic stress signaling, identifying it as a responsive node within metabolism–immune crosstalk. This bidirectional relationship suggests a more integrated model of USP7 function. Under nutrient-rich or tumor-favorable conditions, USP7 may support checkpoint signaling, TAM polarization, lipid stability, and stress tolerance. Under nutrient stress or targeted metabolic intervention, USP7 activity or stability may decline, thereby reducing immunosuppression and exposing metabolic weakness. Such a model helps explain why dietary or metabolic manipulation could potentially synergize with USP7-targeted therapy. Future work will be needed to determine whether other metabolic cues, such as hypoxia, lactate accumulation, amino acid restriction, or mitochondrial stress, also regulate USP7. If so, USP7 may represent a broader stress-responsive regulatory node linking environmental stress to malignant adaptability (**Figure 7**), although this possibility still requires validation across additional tumor contexts. **Table 2** summarizes how USP7 regulates metabolic plasticity, redox homeostasis, and nutrient-stress responses, linking metabolism to immune evasion and therapy resistance.

**Table 2:** USP7-associated metabolic adaptation, redox control, and nutrient stress responses in cancer.

Cancer type/context	Experimental model	Metabolic axis or stress-response pathway	Representative biological effect	Mechanistic implication
Gastric cancer	Cell lines and murine models	SCD-dependent lipid metabolism	Maintains lipid homeostasis and suppresses ferroptosis	USP7 stabilizes SCD, thereby supporting lipid resilience and survival under oxidative stress
Breast cancer	Tumor exosome–macrophage co-culture system	NRF2-dependent redox adaptation	Enhances antioxidant defense and favors M2-like macrophage polarization	USP7 reinforces redox homeostasis while coupling oxidative stress adaptation to immune suppression
Liver cancer	Short-term starvation and murine models	USP7 UFMylation–RBPJ axis under nutrient stress	Links metabolic stress to alleviation of T-cell exhaustion	Nutrient stress reduces USP7 UFMylation, destabilizes RBPJ, and attenuates exhaustion-associated immune dysfunction

## 5 USP7 and therapeutic resistance

### 5.1 USP7 as a driver of resistance to immune checkpoint blockade

Therapeutic resistance is rarely explained by a single pathway, especially in immunotherapy. Tumors evade checkpoint blockade through multiple mechanisms, including low immune infiltration, inadequate antigen presentation, suppressive myeloid populations, compensatory checkpoint pathways, stromal exclusion, and metabolic competition. USP7 is uniquely positioned to participate in several of these mechanisms simultaneously (77–80). Its role in stabilizing PD-L1 directly links USP7 to resistance against PD-1/PD-L1-targeted therapy. Its regulation of FGL1 suggests that tumors with high USP7 activity may also rely on the LAG-3 axis, thereby creating layered checkpoint redundancy. In addition, USP7-mediated TAM polarization, T-cell dysfunction, and suppression of chemokine-driven immune recruitment contribute to a microenvironment in which checkpoint blockade alone may be insufficient. This multi-level influence suggests that USP7 is not simply associated with immunotherapy resistance but may mechanistically sustain it. Findings in microsatellite-stable colorectal cancer and hepatocellular carcinoma are especially informative in this regard. These tumors are often poorly responsive to checkpoint inhibitors, yet USP7 inhibition appears capable of increasing immune-cell recruitment and restoring antitumor immune activity. This supports a model in which USP7 targeting is less a replacement for immunotherapy than a sensitizing strategy that renders resistant tumors more permissive to immune attack.

### 5.2 Microenvironmental remodeling and therapy tolerance

A major limitation of many targeted anticancer agents is that they focus primarily on tumor-cell-intrinsic signaling while failing to address the supportive role of the tumor microenvironment. USP7 appears to overcome this limitation by affecting multiple cellular compartments within tumors. In lung cancer and breast cancer, USP7 shapes macrophage polarization. In colorectal cancer, it affects chemokine networks and lymphocyte recruitment. In additional work, USP7 inhibitors were shown to suppress tumor neoangiogenesis by downregulating fibroblast-derived VEGF, thereby enhancing synergy with immune checkpoint inhibitors (81). This is highly significant because abnormal vasculature and stromal signaling are major contributors to therapy resistance. Poor vessel architecture impairs immune-cell infiltration, creates hypoxic niches, and favors immunosuppressive cell states. Fibroblast-derived factors can further reinforce immune exclusion and treatment tolerance. By lowering VEGF and improving the immune contexture, USP7 inhibition may help dismantle a microenvironmental shield that protects tumors from therapy. Thus, USP7 should be considered a regulator of treatment-refractory niches rather than merely a tumor-cell survival factor. This perspective broadens the rationale for targeting USP7 and suggests that its therapeutic benefit may derive in part from normalizing the ecological conditions that permit resistance.

### 5.3 Metabolic adaptation as a mechanism of treatment tolerance

Metabolic flexibility is a major driver of therapy resistance. Tumors exposed to chemotherapy, radiotherapy, targeted agents, or immune attack often survive by switching metabolic programs, increasing antioxidant defense, and resisting forms of stress-induced cell death. The emerging connection between USP7 and lipid metabolism, redox balance, and ferroptosis indicates that this DUB may help maintain the metabolic robustness required for treatment tolerance (82–86). By stabilizing SCD and supporting membrane lipid homeostasis, USP7 can protect cancer cells from ferroptotic death (87). By reinforcing NRF2-related antioxidant adaptation, it may help tumors survive oxidative and therapeutic stress. By participating in nutrient-sensitive pathways that affect T-cell exhaustion, USP7 may simultaneously weaken immune-mediated elimination. These combined effects imply that therapeutic resistance should be viewed not only as a problem of signaling bypass but also as a state of coordinated metabolic and immunological resilience. This framework has practical implications. Tumors with high USP7 activity may be especially dependent on post-translational maintenance of stress-adaptive programs. In such tumors, USP7 inhibition could collapse both immune suppression and metabolic tolerance, thereby producing a broader therapeutic effect than agents targeting either arm alone.

### 5.4 Determinants of context specificity in USP7 biology and therapeutic response

**Figure 8:**
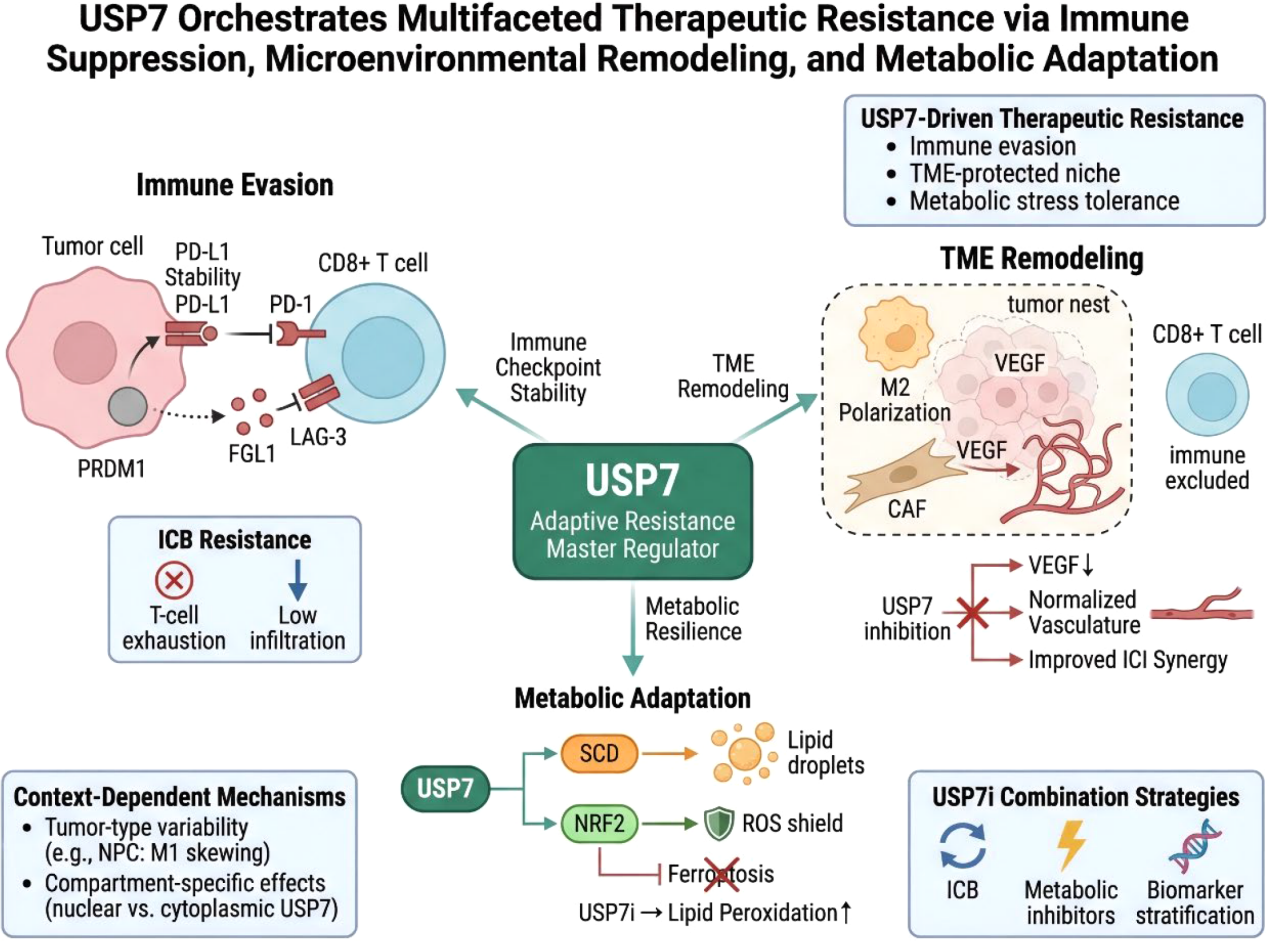
USP7 drives therapeutic resistance through immune suppression, microenvironmental remodeling, and metabolic adaptation. This schematic illustrates how USP7 sustains treatment resistance by stabilizing checkpoint pathways, promoting immunosuppressive and angiogenic tumor microenvironments, and maintaining metabolic stress tolerance through lipid and redox adaptation. These integrated functions position USP7 as a context-dependent hub of malignant adaptability and a potential target for therapeutic sensitization.

Despite the growing evidence supporting USP7 as a regulator of tumor immune evasion, metabolic adaptation, and therapeutic resistance, its biological role is clearly not uniform across tumor types or cellular compartments. In the current literature, USP7 most often exhibits tumor-promoting functions; however, findings such as those in nasopharyngeal carcinoma indicate that its effects may, in selected contexts, diverge from this dominant pattern (23, 88). Rather than treating these observations as isolated exceptions, it may be more informative to view them as evidence that USP7 biology is fundamentally context-dependent. Several factors may underlie this context specificity. First, tumor lineage is likely to be important, because lineage-specific transcriptional and epigenetic programs shape the substrate landscape available to USP7. Second, driver alterations may influence which USP7-regulated pathways become functionally dominant, thereby shifting its major outputs toward checkpoint stability, macrophage education, metabolic stress tolerance, or other adaptive programs. Third, viral association may be relevant in selected cancers such as nasopharyngeal carcinoma, where inflammatory tone and host-pathogen signaling may alter the biological consequences of USP7 activity. Fourth, metabolic state may influence both the availability of USP7 substrates and the stress-adaptive value of USP7-dependent signaling, particularly under conditions of redox imbalance, ferroptotic pressure, or nutrient deprivation (89–91). Fifth, stromal composition and microenvironmental architecture are likely to be critical, because USP7 can act not only in tumor cells, but also through macrophages, fibroblast-associated angiogenic programs, and broader immune ecosystem remodeling. Finally, treatment history may reshape USP7 dependency over time, as prior exposure to chemotherapy, targeted therapy, or immunotherapy could select for tumor states in which particular USP7-regulated pathways become more important. These considerations suggest that context dependence should not be viewed merely as a caveat, but as a central organizing principle for understanding USP7 biology. Some tumors may rely on USP7 primarily for checkpoint stabilization, others for myeloid reprogramming, others for metabolic resilience, and still others for combined ecological support. This framework has important translational implications, because it argues against a one-size-fits-all model of USP7 inhibition and instead supports biomarker-guided, tumor-context-specific therapeutic strategies (**Figure 8**).

## 6 Therapeutic targeting of USP7

### 6.1 Development of USP7-targeted agents

USP7 has attracted growing attention as a drug target because it sits upstream of multiple tumor-promoting outputs. Early inhibitors such as P5091 provided valuable proof-of-concept that USP7 blockade could suppress tumor growth and alter immune responses (92–94). Subsequent medicinal chemistry efforts generated additional small-molecule inhibitors with improved selectivity and pharmacological properties, including catalytic and allosteric inhibitors that helped establish the tractability of USP7 as a therapeutically actionable deubiquitinase (95–98).

More recent studies have further expanded the chemical space of USP7-targeting agents. For example, newly developed 5-amino-pyrazole derivatives were reported as USP7 inhibitors, with several compounds showing dose-dependent enzyme inhibition and improved activity relative to the parental scaffold (99). In parallel, virtual screening and molecular simulation-based approaches have identified additional USP7-directed compounds, including BML-284, which showed antitumor activity in neuroblastoma in association with suppression of the USP7/N-Myc axis (100). These studies indicate that USP7 inhibitor development remains an active and rapidly evolving field, with continued diversification of chemical scaffolds and optimization strategies. Importantly, the pharmacological landscape of USP7 is now extending beyond conventional occupancy-driven inhibition. Structural and functional studies recently identified MS-8 as a small-molecule allosteric activator of USP7, demonstrating that this deubiquitinase can be pharmacologically modulated in more than one direction (101). Although such activators are not anticancer inhibitors per se, they highlight the conformational plasticity and druggability of USP7 and may inform future structure-guided ligand design.

Beyond small-molecule inhibition, targeted protein degradation has also emerged as a promising strategy for USP7 intervention. A recent study described a selective USP7 degrader, D16, designed from a thienopyridine-containing USP7 ligand linked to a CRBN-recruiting moiety. D16 induced dose-dependent degradation of USP7, showed selectivity over other USP family members by proteomic analysis, and impaired migration of upper gastrointestinal cancer cells with limited effects on proliferation (102). These findings suggest that degradation-based approaches may complement catalytic inhibition and broaden the translational toolbox for USP7-directed therapy, particularly in settings where sustained depletion of the USP7 protein may be preferable to transient enzymatic blockade.

Taken together, these advances indicate that USP7-directed drug development is entering a more diversified phase, encompassing classical inhibitors, structurally novel enzyme modulators, and degrader-based strategies. Nevertheless, important challenges remain, including selectivity over related deubiquitinases, context-dependent biological effects, pharmacokinetic optimization, and biomarker-guided patient selection. Addressing these issues will be essential for translating USP7-targeted agents into clinically meaningful oncology strategies. **Table 3** emphasizes translational opportunities of USP7 inhibition, highlighting its multi-faceted effects and potential combinatorial strategies with immunotherapy or metabolism-targeting interventions.

**Table 3:** Therapeutic targeting of USP7: representative inhibitors, biological effects, and combination strategies across cancer contexts.

USP7-targeting agent/strategy	Cancer type/model	Major pathway or biological axis affected	Representative therapeutic effect	Combination potential/translational implication
P5091, Almac4, or pharmacological USP7 inhibition	Lung cancer, gastric cancer, colorectal cancer	PD-L1, FGL1, TAM reprogramming	Reduces immune suppression and enhances antitumor T-cell activity	May synergize with anti-PD-1/anti-PD-L1 therapy
Selective USP7 inhibition	NRAS-mutant melanoma	Immune checkpoint signaling and MEK-associated adaptive resistance	Enhances responsiveness to anti-PD-1 therapy	Rational combination with MEK1/2 inhibitors
USP7 inhibition	Gastric cancer	SCD-dependent lipid metabolism and ferroptosis resistance	Destabilizes lipid homeostasis and induces ferroptosis	May be combined with ferroptosis inducers, oxidative stress-based therapy, or metabolism-targeting agents
USP7 inhibition	Breast cancer	NRF2-dependent redox adaptation and macrophage polarization	Reduces M2-like macrophage-mediated immunosuppression	Potential combination with redox-modulating strategies or immunotherapy
USP7 inhibition	Liver cancer	FGL1/LAG-3 axis and T-cell exhaustion	Restores CD8^+^ T-cell function and alleviates exhaustion-associated suppression	Potential combination with checkpoint blockade and/or nutrient-modulating interventions
USP7 degrader or next-generation modality	Preclinical solid tumor models	Sustained depletion of USP7 protein and multi-pathway suppression	May provide broader or more durable pathway inhibition than transient catalytic blockade	Supports future expansion toward degrader-based and biomarker-guided therapeutic development

### 6.2 USP7 inhibition as a strategy to enhance immunotherapy

A major strength of USP7 targeting is its ability not only to cooperate with immune checkpoint blockade, but also to enhance the efficacy of immunotherapy more broadly. In lung cancer, USP7 inhibition enhances antitumor immune responses in a macrophage-dependent manner and shows synergy with PD-1 blockade (46). In microsatellite-stable colorectal cancer, USP7 inhibition converts immune-cold tumors toward a more inflamed phenotype and improves anti-PD-1 efficacy (50). In NRAS-mutant melanoma, selective USP7 inhibition also synergizes with MEK1/2 inhibition to potentiate anti-PD-1 therapy (103). These studies collectively suggest that USP7 inhibition may function as an immunotherapy-sensitizing strategy rather than merely as a standalone anticancer intervention. Mechanistically, USP7 inhibition may enhance immunotherapy efficacy by lowering checkpoint ligand abundance, reducing myeloid-mediated immunosuppression, improving immune-cell infiltration, and weakening stromal barriers that limit effective antitumor responses. Through these combined effects, USP7 inhibition has the potential to address several distinct failure points of immunotherapy simultaneously. This systems-level action may be particularly valuable in tumors that do not respond to checkpoint blockade because of layered resistance mechanisms rather than isolated checkpoint dependence.

### 6.3 Combination strategies with targeted and metabolism-oriented therapy

Beyond checkpoint inhibitors, USP7 blockade may be combined with targeted therapies and metabolic interventions. The synergy observed with MEK1/2 inhibitors in melanoma suggests that USP7 inhibition can cooperate with oncogenic pathway blockade to produce enhanced immune benefit. This raises the possibility that other pathway-directed agents may also pair effectively with USP7 inhibitors, particularly where oncogenic signaling contributes to immune suppression or metabolic reprogramming. The connection between USP7 and ferroptosis further suggests a rationale for combining USP7 inhibition with therapies that increase oxidative stress or disrupt lipid metabolism. Such strategies may be especially effective in tumors that use lipid remodeling and antioxidant defenses to escape cell death. Similarly, nutrient-modulating interventions or AMPK-activating strategies might alter USP7 biology in ways that complement pharmacological inhibition (104). Taken together, these findings suggest that the future of USP7-directed therapy will likely lie in rational combinations rather than monotherapy. The enzyme’s broad regulatory reach makes it well suited to act as a central lever in multi-pronged treatment strategies.

### 6.4 Biomarkers and patient selection

For USP7-targeted therapies to be clinically effective, robust biomarkers will be required to identify tumors that are functionally dependent on USP7-regulated programs. Because USP7 exerts context-dependent effects across tumor cells and the microenvironment, expression of USP7 alone is unlikely to be sufficient as a predictive marker (105–107). Instead, biomarker development should focus on defining USP7 dependency at multiple levels. Potential biomarker categories may include tumor-intrinsic indicators, such as high USP7 expression and elevated checkpoint-related molecules including PD-L1 or FGL1; microenvironmental features, such as immune-cold transcriptional signatures, tumor-associated macrophage-enriched niches, and stromal VEGF-related phenotypes; and metabolic or stress-adaptation features, including signatures associated with ferroptosis resistance or oxidative adaptation (47, 50). These parameters may help identify tumors in which USP7 supports both immune suppression and metabolic resilience. Accordingly, future patient selection strategies will likely require integrated rather than single-marker models, combining transcriptomic, proteomic, spatial, and functional information. Tumors characterized by coexisting checkpoint activation, suppressive myeloid contexture, and marked metabolic plasticity may represent the subgroup most likely to benefit from USP7 inhibition. Such a biomarker-guided framework would improve patient stratification and may help avoid the indiscriminate use of USP7-targeted agents in biologically unsuitable settings.

## 7 Challenges and future perspectives

Although emerging literature supports USP7 as a multifaceted regulator of cancer progression, the evidential maturity of its proposed functions is not uniform, and several important questions remain unresolved. One key issue is substrate prioritization. USP7 regulates many proteins, but it is still unclear which substrates dominate in specific tumor and microenvironmental contexts. Without this information, it is difficult to predict when USP7 inhibition will primarily affect checkpoint signaling, macrophage polarization, metabolic adaptation, or other processes. A second challenge is cell-type specificity. Most current studies focus either on tumor cells or on one immune component at a time, yet the biological action of USP7 likely reflects coordinated effects across several compartments. Single-cell sequencing, spatial transcriptomics, and proteogenomic approaches will be essential for mapping USP7-dependent networks at ecosystem resolution. These techniques may reveal whether USP7 functions as a tumor-cell-intrinsic driver, a stromal modulator, a myeloid regulator, or all of these simultaneously in different cancers. A third unresolved area concerns the integration of metabolism and immunity. Existing studies already suggest that USP7 links lipid homeostasis, oxidative adaptation, nutrient stress signaling, and immune dysfunction. However, the broader scope of these interactions remains largely unexplored. It will be important to determine whether USP7 also interfaces with hypoxia signaling, lactate metabolism, amino acid competition, mitochondrial remodeling, or other metabolic programs known to shape the immune microenvironment. Finally, translational progress will depend on overcoming pharmacological and biological challenges. More selective inhibitors, deeper toxicity profiling, and rational biomarker strategies are needed (107–110). At the same time, context-dependent or paradoxical effects of USP7 must be clarified before widespread clinical development can be justified. Rather than undermining enthusiasm, these complexities should motivate more nuanced models of USP7 biology and more sophisticated therapeutic frameworks.

## 8 Conclusion

USP7 has emerged as far more than a classical deubiquitinase involved in oncogenic protein stabilization. Current evidence suggests that USP7 may function as a multifaceted regulator of tumor immune evasion, metabolic adaptation, and therapeutic resistance in a context-dependent manner. By stabilizing immune checkpoint-associated molecules, reshaping macrophage behavior, influencing T-cell dysfunction, modulating chemokine landscapes, sustaining lipid and redox homeostasis, and supporting treatment-refractory microenvironments, USP7 contributes to several of the core adaptive strategies that define malignant progression. This convergence makes USP7 particularly attractive as a therapeutic target. Unlike pathways that regulate only one aspect of tumor biology, USP7 sits at an interface where tumor-cell-intrinsic survival mechanisms meet extrinsic immune and metabolic constraints. Consequently, targeting USP7 may offer a means to destabilize multiple malignant programs at once, especially when integrated with checkpoint blockade, targeted therapy, or metabolism-directed intervention. At the same time, the biology of USP7 is clearly context-dependent, and its clinical exploitation will require careful patient stratification and deeper mechanistic understanding. Even so, the available evidence suggests that USP7 may represent an emerging mechanistic link in malignant adaptability, although this broader integrative role still requires further validation. Continued investigation of this enzyme may not only refine our understanding of tumor progression but also open new routes toward more effective and durable cancer therapy. Taken together, this review advances a conceptual shift from viewing USP7 as a classical deubiquitinase with isolated substrates to recognizing it as an integrative regulator of tumor immunity, metabolic adaptation, and therapeutic resistance.
